# Cases Cured by Psorinum

**Published:** 1893-02

**Authors:** I. Dever

**Affiliations:** Clinton, N. Y.


### CASES CURED BY PSORINUM.

I. Dever, M. D., Clinton, N. Y.

February 10th, 1889, I was called to see R——, a bright-eyed little boy three years old.

His parents were robust Irish Americans, in whom there was not the least appearance of scrofula, but the child had inherited a degree of vitality far below that of either of his parents.

He had become the subject of a previous attack of illness, which the physician diagnosed as capillary bronchitis, from which he had to all appearance recovered, and remained in good health up to January, 1890, when he again fell sick. The same physician who had attended him through the previous attack was called, as the mother remarked, "To put him through again." Capillary bronchitis was the diagnosis, followed by a course of external and internal medication which left nothing to be desired in the way of heroic treatment, but it left the little patient in what the parents and friends supposed to be a dying condition.

When I was called I found the child covered with flannel and the room heated to an unendurable temperature, in explanation of which I was informed that the patient could not bear the least draught of cold air. He could get no rest except when covered, head and eyes, with warm flannel, and then he would only be quiet for ten or fifteen minutes at a time.

His favorite position was with his head and face pushed deep into the pillow. At first I thought he assumed this position to exclude the light, as his eyes were red and inflamed, nevertheless I regarded it as a characteristic, and on further investigation found it one of significant importance when I came to make choice of the curative remedy.

The fever had been running for three weeks and the child was terribly emaciated, the abdomen was distended, and there was diarrhœa, the character of which was thin and offensive.

He had a cough with a moist bronchial rattle caused by a collection of mucus in the trachea and bronchial tubes. There was dyspnœa when the patient was held in a sitting position. Great drops of perspiration covered the face and upper lip. I had no thermometer with me, but I found the pulse to be 150 and the respiration 50 in a minute.

Auscultation revealed the fact that the bronchia and large air cells were loaded with a mucus so tough that it could not be expectorated. The patient when well was of a good disposition, but now he kept up a constant whine, and was so peevish that he would cry or whine when spoken to or touched though ever so gently. After my examination of the child I told the father that I would much rather not have been called at so late a stage of the case, but I would prescribe, providing he would allow me to treat the boy to the end, to which he agreed, as he had no hope of his recovery, unless relief came soon.

Now for a prescription.

The symptoms on which to base a homœopathic prescription were somewhat in the background, from the fact that the child had been so thoroughly medicated with everything except the right thing that it was difficult to tell just which symptoms were caused by the super-abundant medication and which were purely the result of the cause which had so disturbed the vital-forces that life appeared to be hanging in a balance. However, after thinking the matter over I prescribed Tart-emet.$^{200}$, and waited for five days, at the expiration of which time I could see no change further than I could attribute to a cessation of the large quantity of allopathic medicine he had been compelled to swallow. I thought I could see Sulphur as the indicated remedy and prescribed one dose, 200, but no change came about for the better. The child had eaten nothing since his illness, he had not been free from fever, and he coughed all the time. I could not see that he was less sensitive to the cold, and the perspiration still stood out on his upper lip. All who saw him would say, "Poor little boy, he is going with consumption."

To all appearances this was a hopeless case, and all had given up except the mother and myself. And I must confess that so far my prescriptions had disappointed my expectations. But the patient was yet alive, and with a firm faith in the law of similars I wrote out another description of the case, which directed my attention to Psorinum. Here the symptoms are: The boy hides his face in the pillow, and finds his only relief by lying on his face. He is covered with perspiration, but more especially on the face and upper lip. His eyes are inflamed and he cannot bear the light. He has pains in his ears after being exposed to cold air. He cannot bear the cold but wants the head covered up warm. Has thirst, drinks often, but little at a time. Coughs and rattles all over the chest, which can be distinctly heard by placing the ear to the thorax. Abdomen distended. Dyspnœa and palpitation of the heart. One dose of Psorinum$^{1500}$ greatly aggravated the symptoms and I was called in a hurry, but I did not interfere with the action of my remedy but allowed this one dose to complete the case, which has remained good up to date.

Case second. Miss E——, a maiden lady of thirty-eight summers, called at my office September 9th, 1888.

The burden of her complaint was a sore head which dated back to the time of her childhood. She had never been treated by a homœopath and could tell but little about herself, except she had a sore head, and, like many who come to us for relief, thought that that was enough for the doctor to know. She finally exhibited some spots on her arms, which she said itched and burned when scratched. Her head was covered with dirty scabs, which exuded a dirty, sticky, bad-smelling serum that matted her hair together in one tangled mass. I prescribed one dose of Sepia$^{200}$, but missed the mark. She called in a year to tell me that my medicine did not help her. I then prescribed Psorinum$^{200}$, one dose, which aggravated her sufferings and brought the eruption out on her face and ears, until she was a "sight," as she expressed it, and to get relief she called on another physician who prepared her a tar ointment, but she was afraid to use it and sent to me for more medicine. I sent Psorinum$^{1500}$, one dose, which cured the difficulty, and the cure still remains good to the present writing.

August 9th, 1891, Mrs. W—— called me to see her nine-months-old baby. This baby had been treated for three weeks by an allopathic doctor for what he called cholera infantum, and of course I found it under the influence of a narcotic. The head was thrown back, eyes half open, pupils contracted, and other indications of scientific treatment. The temperature was 105°, the pulse so weak and fast that I could not count it at the wrist.

The mother told me that her baby had been in the above-named condition for over three weeks. The diarrhœa was thin and of a dirty brown color, voided in a gush, and very offensive. I could not examine the discharges, as they had to be removed and thrown away on account of their fetid condition. Judging from her description of the discharges I gave Pod.$^{200}$. Called next day to find the narcotic symptoms had all disappeared, but there was little, if any, change in respect to the diarrhœa. No medicine for some days until I could gather symptoms on which to make a homœopathic prescription.

This was a hand-fed child. I substituted one part of fresh cream to two parts of hot water, to which I added a little sugar in the place of a diet of milk. After carefully writing out the important symptoms I had the following disposition : Peevish, whining, cross, with no disposition to sleep. Will not sleep except on the face. Head hot and perspiring ; covered with perspiration. He vomited everything taken into the stomach. Abdomen was distended. The diarrhœa was thin, dirty brown, and fetid, smelling like carrion. *Worse* at night. *Thin,* containing green mucus mixed with blood. Cold aggravated all of the symptoms. I regard the peevish disposition, the inclination to perspire, the position taken by the child with its face bored deep into the pillow, together with the sensitiveness to cold as characteristic of Psorinum, and gave a dose, 200, which cured the vomiting and diarrhœa, but brought out a crop of dark blue pustules, which covered the little patient's head and breast. But the child grew better right along and recovered without further medicine, and to-day he is a bright, healthy boy, and may some day be elected President for aught I know.

